# 2599. Impact of Alveolar Neutrophilia on Outcomes in Patients with Suspected Ventilated Hospital-Acquired Bacterial Pneumonia

**DOI:** 10.1093/ofid/ofad500.2214

**Published:** 2023-11-27

**Authors:** Louis Saravolatz, Owen Albin

**Affiliations:** Michigan Medicine, Ann Arbor, Michigan; University of Michigan Medical School, Ann Arbor, MI

## Abstract

**Background:**

Ventilated hospital-acquired bacterial pneumonia (vHABP) is a major source of morbidity & mortality in hospitalized patients. Clinical risk scores and systemic biomarkers have limited clinical & prognostic utility in vHABP. Alveolar biomarkers—specifically alveolar neutrophilia (AN), the percentage of polymorphonuclear cells (PMN%) present in bronchoalveolar lavage (BAL) fluid—gauge the host inflammatory response directly at the site of infection, offering an attractive but untested predictive risk biomarker in vHABP. We aimed to generate preliminary data exploring AN as a vHABP biomarker.

**Methods:**

We performed a retrospective cohort study of intensive care unit (ICU) patients with vHABP from 2010-2023. vHABP was defined as development of acute hypoxic respiratory failure requiring invasive mechanical ventilation, clinical suspicion of pneumonia by treating teams prompting performance of BAL respiratory culture, and BAL PMN% > 50%. Patient demographic characteristics, comorbidities and acute severity of illness metrics were abstracted via chart review. PMN% values were dichotomized using median split rounded to the nearest PMN% decile ( > or < 80% PMN% in BAL fluid). Study outcomes included ventilator-free days, ICU length of stay and vHABP-directed antibiotic treatment duration within 30 days of vHABP.

**Results:**

44 patients met study inclusion criteria. 30 (68%) had a BAL neutrophil of > 80%. Patients with BAL PMN > 80% had a numerically higher use of tracheostomy and trend towards lower P/F ratio at the time of vHABP diagnosis [Table 1]. BAL PMN > 80% was associated with a 15% decrease in ventilator-free days (p < 0.01) and a 25% longer ICU length of stay (p < 0.01) than those with BAL PMN < 80% [Figure 1]. There was no significant difference in post-BAL vHABP-directed antibiotic duration in patients with and without BAL PMN > 80%.

**Figure:**
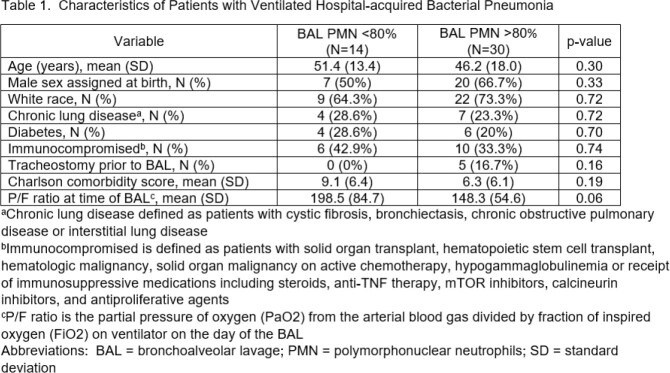

**Figure:**
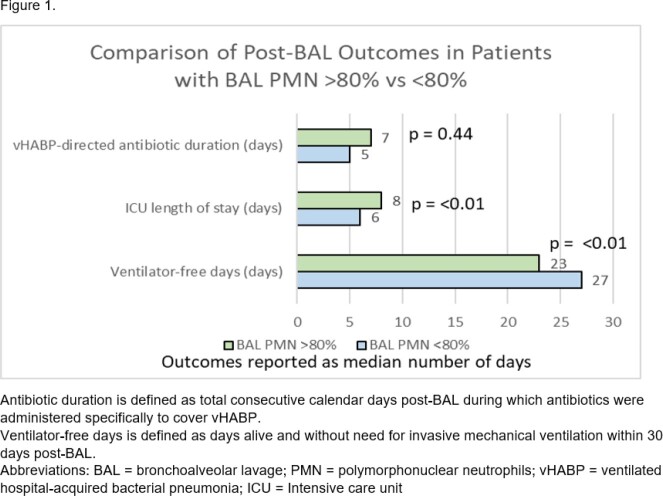

**Conclusion:**

Though limited by low sample size, this exploratory study suggests AN may be a potentially useful predictive risk biomarker in vHABP. Larger-scale, prospective studies of alveolar biomarkers—including AN—in vHABP are warranted.

**Disclosures:**

**Owen Albin, MD**, Charles River Laboratories: Advisor/Consultant|Shionogi: Advisor/Consultant

